# An analysis of benign human prostate offers insights into the mechanism of apocrine secretion and the origin of prostasomes

**DOI:** 10.1038/s41598-019-40820-2

**Published:** 2019-03-14

**Authors:** Nigel J. Fullwood, Alan J. Lawlor, Pierre L. Martin-Hirsch, Shyam S. Matanhelia, Francis L. Martin

**Affiliations:** 1 Biomedical and Life Sciences, Faculty of Health and Medicine, Furness College, Lancaster University, Lancaster, LA1 4YG UK; 2 Centre for Ecology and Hydrology, Lancaster Environment Centre, Library Avenue, Bailrigg, Lancaster, LA1 4AP UK; 3 Sharoe Green Lane North, Royal Preston Hospital, Fulwood, Preston, PR2 9HT UK; 4 School of Pharmacy and Biomedical Sciences, University of Central Lancashire, Preston, PR1 2HE UK

## Abstract

The structure and function of normal human prostate is still not fully understood. Herein, we concentrate on the different cell types present in normal prostate, describing some previously unreported types and provide evidence that prostasomes are primarily produced by apocrine secretion. Patients (*n* = 10) undergoing TURP were prospectively consented based on their having a low risk of harbouring CaP. Scanning electron microscopy and transmission electron microscopy was used to characterise cell types and modes of secretion. Zinc levels were determined using Inductively Coupled Plasma Mass Spectrometry. Although merocrine secretory cells were noted, the majority of secretory cells appear to be apocrine; for the first time, we clearly show high-resolution images of the stages of aposome secretion in human prostate. We also report a previously undescribed type of epithelial cell and the first ultrastructural image of wrapping cells in human prostate stroma. The zinc levels in the tissues examined were uniformly high and X-ray microanalysis detected zinc in merocrine cells but not in prostasomes. We conclude that a significant proportion of prostasomes, possibly the majority, are generated *via* apocrine secretion. This finding provides an explanation as to why so many large proteins, without a signal peptide sequence, are present in the prostatic fluid.

## Introduction

There are many complications associated with the prostate from middle age onwards, including benign prostatic hyperplasia (BPH) and prostate cancer (PCa). These contribute significantly to morbidity and mortality in older men^1^. As a result, there is a vast literature on the diagnosis and treatment of prostatic diseases. In comparison, relatively little work has been carried out on the functioning of the normal prostate^2,3^. In particular, there is a surprising lack of agreement on the cell types present in this gland and their precise roles.

The prostate gland is a walnut-shaped gland found in the human male, which surrounds the urethra and the neck of the urinary bladder^4^. It can be broadly subdivided into three different zones, which have been named based on their morphology and appearance. These three zones are the central zone (CZ), peripheral zone (PZ) and transition zone (TZ). The PZ and TZ are believed to originate from endoderm whereas the CZ is believed to derive from the ectoderm^5^. The PZ makes up about 70% of the gland and is the region often considered most prone to PCa. This region is also the easiest to examine and detect any abnormalities. The CZ comprises about 25% of the gland comes next and is rarely associated with carcinogenesis^6^. Finally, the TZ is the region of the gland closest to the urethra; it comprises only 5% of the prostate gland. However, because of its proximity to urethra any enlargement of this region impacts directly on urethral function.

As an endocrine gland, the prostate is involved in the metabolism of testosterone into a more effective androgen. It is also an exocrine gland and is responsible for the secretion of prostatic fluid that is enriched with proteins, enzymes, lipids, metal ions and amines, which makes seminal fluid slightly acidic. Prostatic fluid plays a role in protecting the sperm by reducing the acidity of the urethra, facilitating and enhancing sperm motility. Moreover, the prostatic acidic phosphatase is involved in the nutrition of the spermatozoa by hydrolysing phosphorylcholine into choline. The prostate gland is also responsible for the secretion of high levels of zinc, which is believed to contribute to sperm viability^7,8^.

There remains disagreement as to the precise roles of the epithelial cells present in the glandular prostate. It is generally accepted that the cells in the glandular elements are in a stratified or pseudo-stratified system and that small, undifferentiated basal cells are found in the basal membrane of each glandular element. Sitting above these will be the secretory or luminal cells. The luminal/secretory cells are responsible for producing the components of the prostatic fluid, which is extremely complex containing many hundreds of different molecules; many of these are large proteins without conventional peptide signal sequence. These cells need high levels of testosterone for their survival^9^. The secretory component is derived from both merocrine and apocrine cells; however, there is little or no agreement as to the precise roles of the apocrine secretory cells present, what they contribute to prostatic fluid, and most puzzlingly of all, why both merocrine and apocrine secretory cells are necessary. The basal cells are non-secretory; also, they are morphologically distinct with a low nucleus-to-cytoplasmic ratio characteristic of stem cells. These basal cells are androgen-independent although they may respond to androgen stimuli. It is believed that all or a subpopulation of these basal cells act as progenitor or stem cells for the prostate. Finally, neuroendocrine cells are also present in low numbers. The stroma of human prostate has been relatively neglected at the ultrastructural level. It is known that it consists of bundles of collagen fibrils with a scattering of fibroblast cells, smooth muscle cells, blood vessels and nerves. Although several different sub-types of stromal cells have recently been reported in mouse^10^, these have not yet been identified in human.

The aim of this study was to look in detail at the different cell types present in normal prostate and to try to better elucidate their specific roles with particular attention to the structure and role of apocrine cells. We describe an ultrastructural study using scanning electron microscopy (SEM) and transmission electron microscopy (TEM). SEM has the advantage of allowing the user to examine large volumes of tissue rapidly and provides an excellent understanding of the overall 3-D architecture of the tissue. TEM by contrast is slower and only a tiny fraction of the tissue can be examined, but of course, it does provide unparalleled resolution. Although there have been several excellent ultrastructural reports in the past, these were published several years ago and subsequently our knowledge of the prostate has increased greatly. This study has been carried out and discussed in the context of the most up to date understanding of the structure and function of the human prostate.

## Materials and Methods

### Ethical Approval

Statutory approval for collection of prostate tissue for research was obtained from the Preston, Chorley and South Ribble Local Research Ethics Committee (LREC Approval No. 06/Q1309/76). This study was conducted according to the principles of the Declaration of Helsinki and all other applicable national or local laws and regulations. All patients gave written informed consent before any protocol-specific procedure was performed. Patients (*n* = 10) undergoing TURP were prospectively consented on the basis of their having a low risk of harbouring CaP (no previous history of CaP, benign-feeling gland on digital rectal examination and prostate-specific antigen (PSA) <10 ng mL^−1^ serum); in all cases, histology was classed as benign. All samples in the study came from the transition zone (TZ).

### Samples

For electron microscopy, samples were placed in 4% glutaraldehyde in phosphate buffered saline (PBS) at 4 °C. For Inductively Coupled Plasma Mass Spectrometry, samples were cut into pieces using a blade; then snap-frozen in liquid nitrogen prior to storage at −85 °C.

### Scanning Electron Microscopy (SEM)

Samples were removed from fixation in 4% glutaraldehyde in PBS, washed 3 times in PBS and placed post-fixation in 2% osmium tetroxide for 1 h. The samples were then washed 3 more times in PBS before being dehydrated through an alcohol series and then transferred to hexamethyldilizane (HMDS; Agar Scientific, UK) for two 30-min changes. Samples were then left overnight to allow the HMDS to sublimate off. In order to obtain optimum images of the interior of the prostate many of the samples were dry-fractured after processing. For imaging samples were mounted on cylinder stubs (Agar Scientific, UK), sputter-coated with gold using an Edwards S150A sputter coater for 4 minutes and examined in a JEOL 5600 SEM. Samples for energy dispersive X-ray microanalysis were sputter coated for just 30 sec. Analysis was carried out with a JEOL JSM-7800F high performance Field Emission SEM fitted with a high performance X-ray Energy Dispersive Spectrometer (X-Max50) with a large area 50 mm^2^ Silicon Drift Detector (SDD) from Oxford Instruments. The prostasomes and merocrine cells were analyzed for zinc *in situ* within the prostate by focusing the electron beam on individual prostasomes or merocrine cells and collecting the characteristic X-rays produced. Analysis of the X-ray counts was carried out with Aztec version 3.0 (Oxford Instruments, UK).

### Transmission Electron Microscopy (TEM)

Samples were removed from fixation in 4% glutaraldehyde in PBS, washed 3 times in PBS and placed post-fixation in 2% osmium tetroxide for 1 h. The samples were then washed 3 more times in PBS before dehydration through an alcohol series. The samples were then transferred to propylene oxide (Agar Scientific, UK) for two 30-min changes. Then a 50/50 mixture of propylene oxide and araldite resin CY212 (Agar Scientific, UK) was left for 6 h before being transferred to 100% resin and left overnight. After that, resin samples were placed in an oven to be polymerized in 60 °C for 24 h. Ultrathin sections were cut on a Reichert E ultramicrotome and stained with Uranyl Acetate and Lead Citrate before examination on a JEOL 1010 TEM.

### Inductively Coupled Plasma Mass Spectrometry (ICPMS)

Tissue samples were prepared using a CEM MARS Xpress pressurised microwave digestion system by heating at 200 °C for 15 min with 10 ml of ultrapure nitric acid (Baker Ultrex II). The tissue digests were diluted 100-fold with ultrapure water (Millipore, >18.2 MOhm) and then zinc was measured using a Perkin Elmer Nexion 300D ICPMS. Internal standards gallium, indium and rhenium were used to compensate for sample matrix effects and any drift in instrument sensitivity during the ICPMS analysis. Two certified reference materials (CRMs), a pig kidney (BCR 186, EU IRMM) and a dogfish liver (DOLT-4, NRC Canada), were used to verify the zinc determinations. A comparison with the certified values for these CRMs shows good agreement with our determinations, *i.e*., for BCR 186 and DOLT-4 we found 119 and 109 mg/kg zinc respectively; certified values were 128 and 116 mg/kg zinc, respectively.

## Results

**Figure 1:**
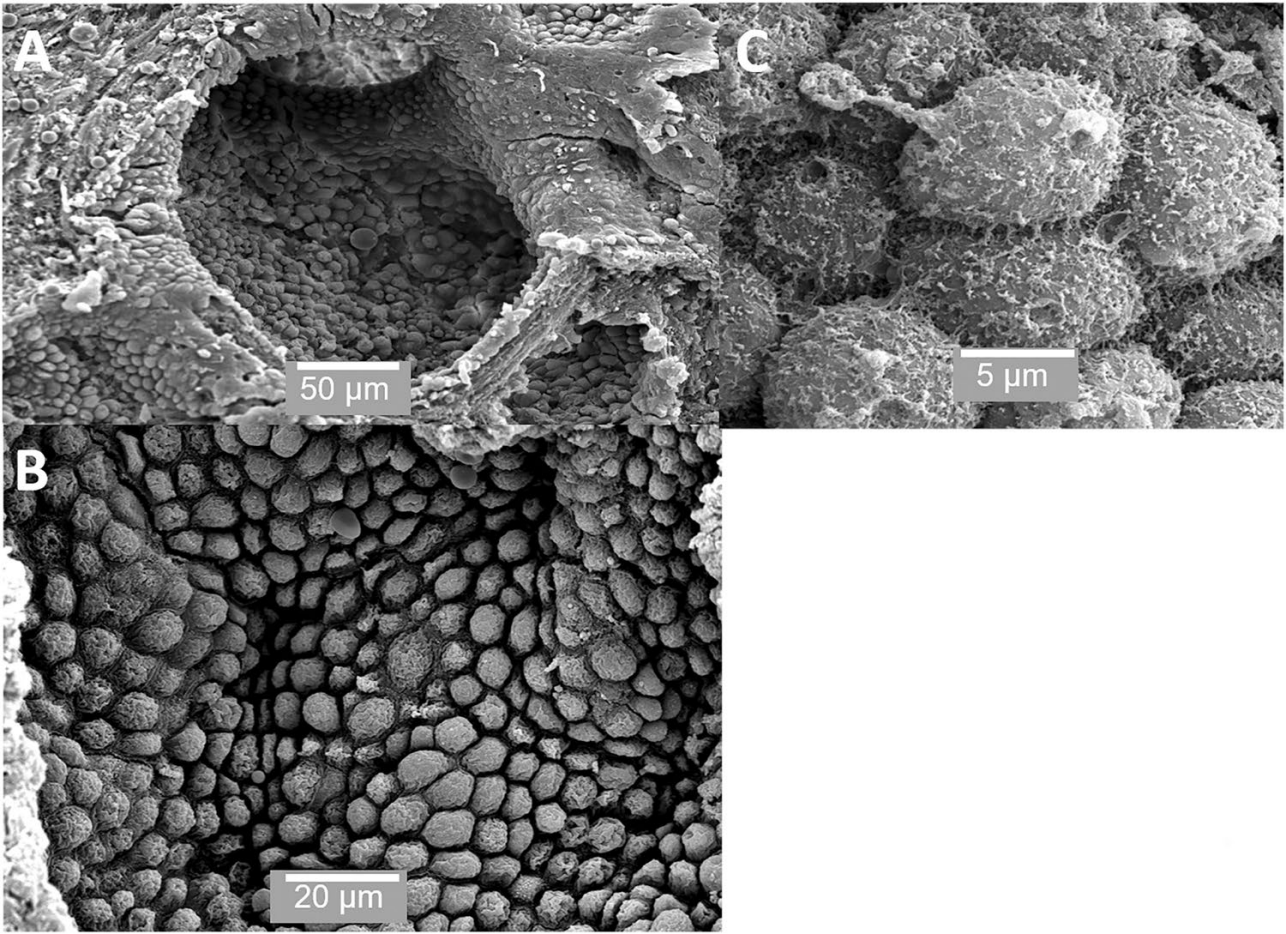
SEM of prostate. (**A**) shows an individual acinus; (**B**) shows the considerable variation in the size of the cells from 10 μm to 5 μm and (**C**) shows the surface of individual cells. Scale bars: A - 50 µm, B - 20 µm, C - 5 µm.

**Figure 2:**
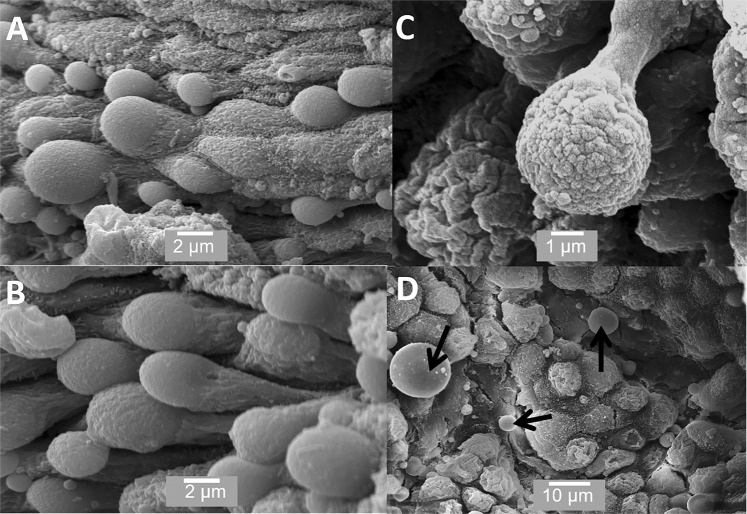
SEM of prostate showing cells undergoing apocrine secretion. (**A**,**B**) shows the apical surface of the cells to consist of rounded dome-like structures; (**C**) shows the budding off process; (**D**) shows completely detached membrane-bound aposomes (arrows), some over a micron in size. Scale bars: A - 2 µm, B - 2 µm, C - 1 µm, D - 10 µm.

**Figure 3:**
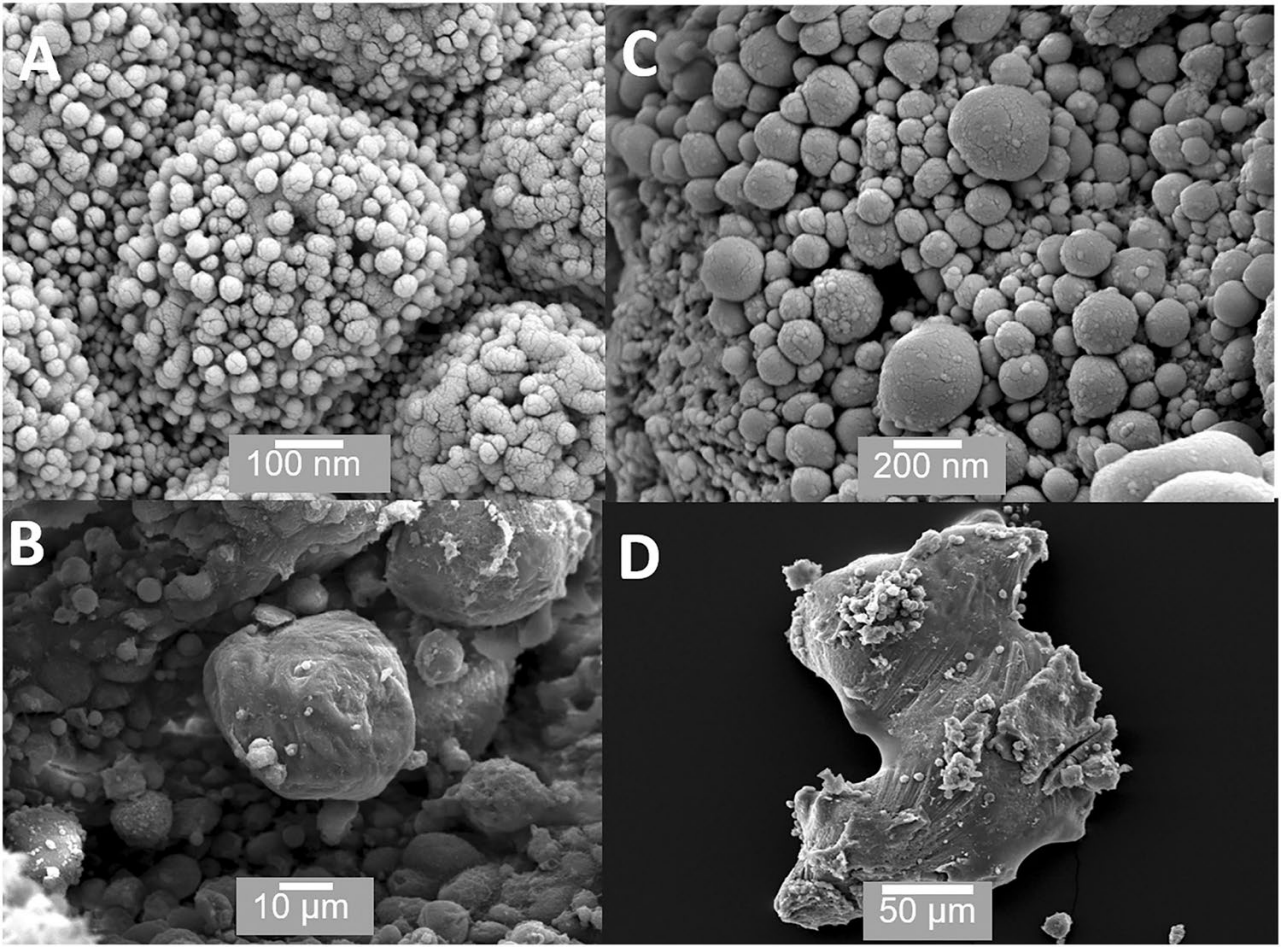
SEM of material found within acini. (**A**) Small (<50 nm) vesicles or exosomes are sometimes visible on the surface of cells; (**B**) shows a small rounded prostatic calculi; (**C**) shows the interior of an acinus packed with prostasomes ranging in size from 150 nm to several hundred nanometres; (**D**) shows an irregular prostatic calculi >200 μm in size (**D**). Scale bars: A - 100 nm, B - 10 µm, C - 200 nm, D - 50 µm.

**Figure 4:**
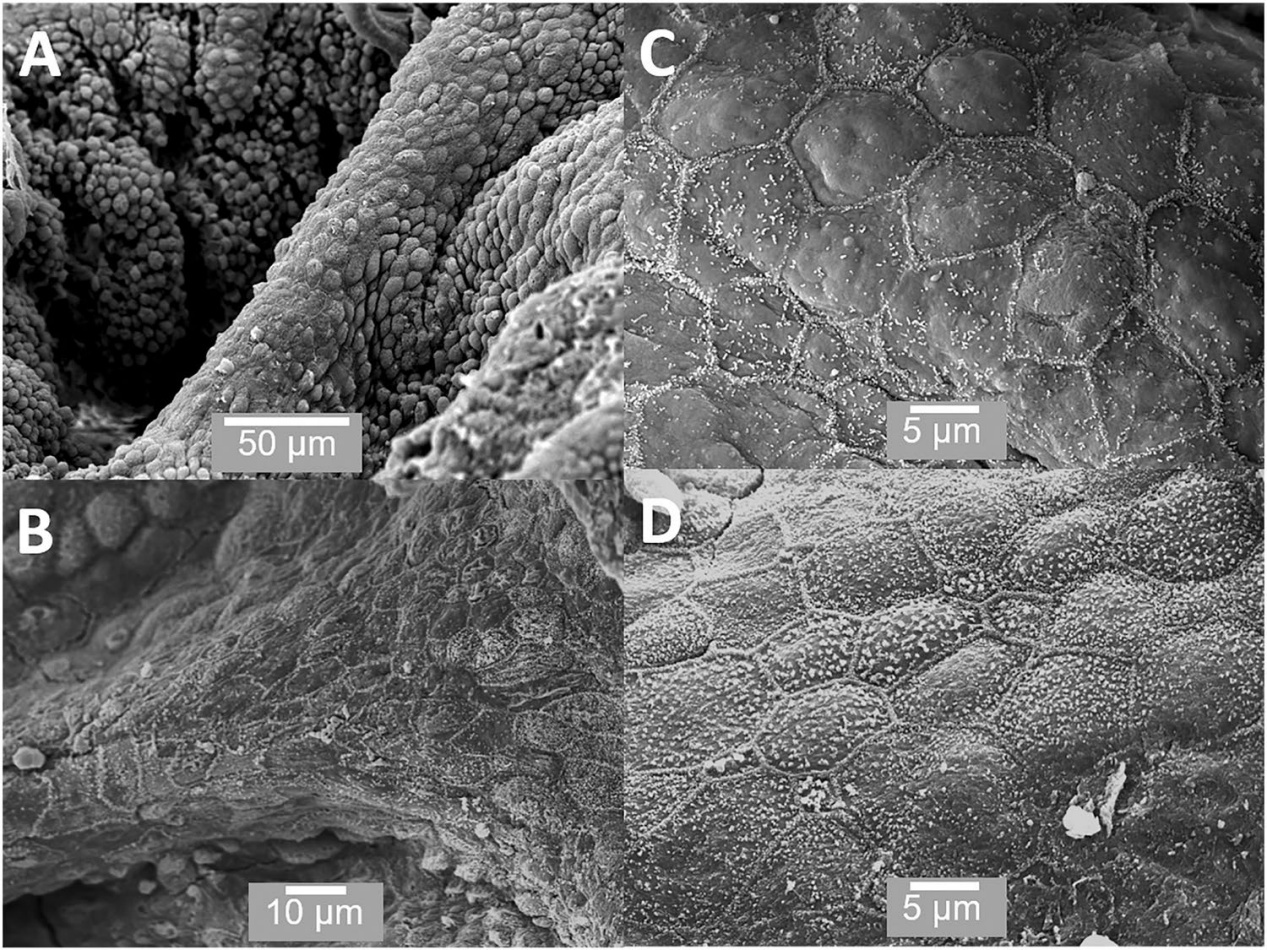
SEM of the ridge-like structures, which separate adjacent acini. (**A**,**B**) The cells running along the ridge-like structures appear different to those within the acini; and, (**C**,**D**) shows the cells to be flat and polygonal with very clearly defined borders. Scale bars: A - 50 µm, B - 10 µm, C - 5 µm, D - 5 µm.

The mature prostate is surrounded by a sheath or capsule of fibroelastic tissue and can be divided into several lobes. Each lobe has many acinar or alveolar structures lined with secretory cells, which empty into ducts within the prostate. Figures 1–4 show SEM images of the prostate. Figure 1 shows these alveolar structures; Figure 1A shows an individual alveolus or acinus, which is packed with secretory cells Figures 1B,C shows the cells in the acinus at medium and high magnification. The secretory cells within individual acini have a variety of appearances and there is considerable variation in the size of the cells. Some of the cells have microvilli on their apical surface Figure 1C. A significant proportion of the cells in the prostate are clearly undergoing apocrine secretion. Figure 2 shows cells undergoing the various stages of apocrine secretion. The apical surface of many of the cells consists of rounded dome-like structures, which are in the process of budding off (Figures 2A,B). Figure 2C also shows an apocrine cell at the stage just before release of the aposome where the aposome is being”pinched off” by the formation of a narrow neck-like region. We observed completely detached membrane-bound structures or aposomes of a variety of diameters. Herein, we use the term prostasome for aposomes >150 nm. Figure 2D shows one very large and several smaller prostasomes that are a visible within one of the acini. This observation was consistent in all the tissues samples we examined, and as far as we are aware, has not been previously reported.

Figure 3 shows the contents found within acini. These include small vesicles on the surface of some cells (Figure 3A). Figure 3B shows a small prostatic calculi (<50 µm); Figure 3C shows an acinus packed with prostasomes. Figure 3D shows large irregularly shaped prostatic calculi over 200 µm in size. Figures 4A,B show the ridge structures between the acini. These structures are interesting in that the cells in these regions are previously unreported and are clearly different from the secretory cells within the acinar sacs. Figures 4C,D show that these cells are polygonal, flat with minimal microvilli and with very clearly defined cell junctions; they do not appear to be secretory.

**Figure 5:**
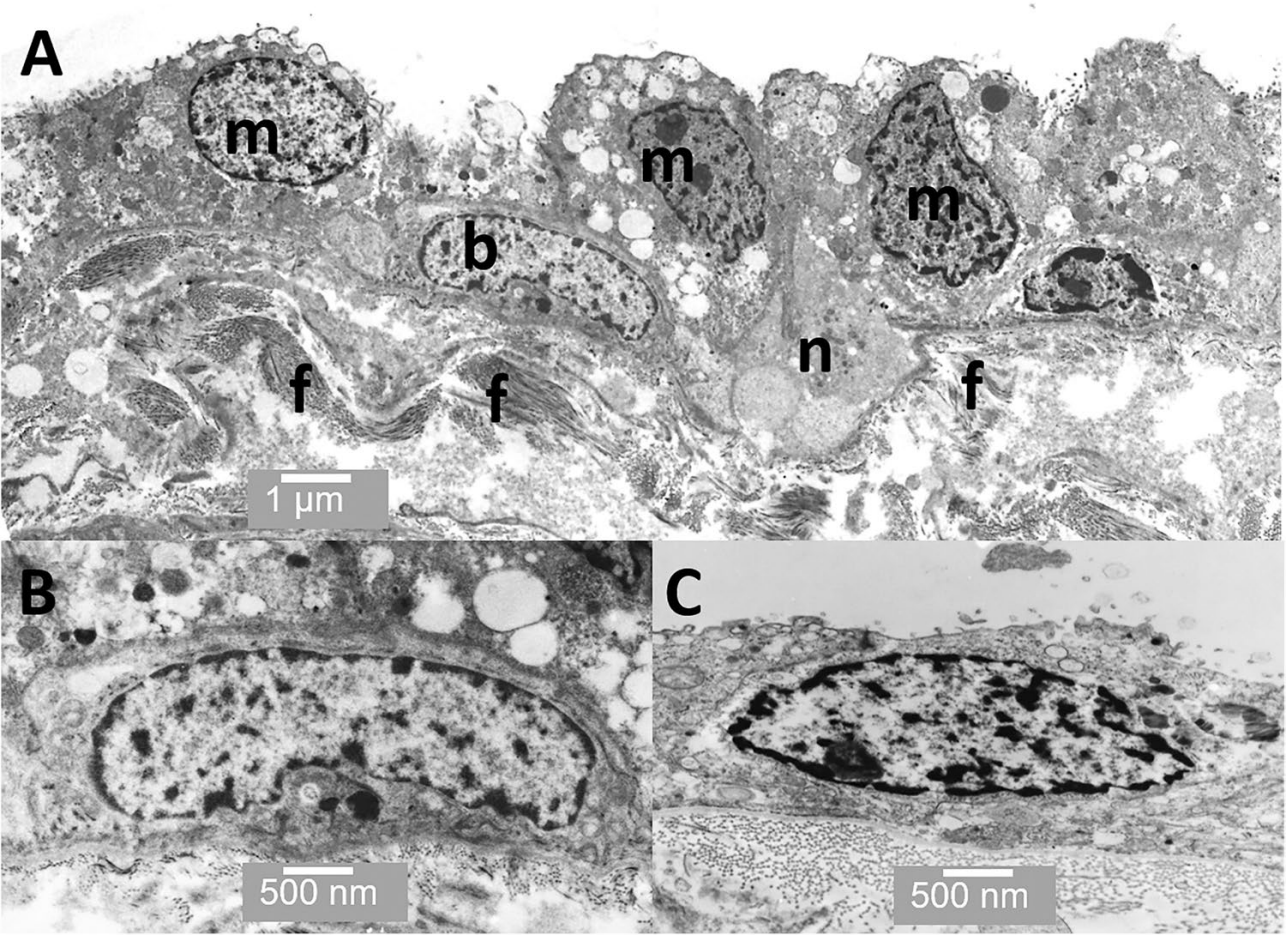
TEM through an acinus. (**A**) Several merocrine secretory cells are evident (m) as well as a basal cell (b) and a neuroendocrine cell (n). Collagen fibrils are also visible (f) (**B**) shows a basal cell in more detail; and (**C**) shows a cuboid epithelial-like cell from the ridge region between the acini. Scale bars: A - 1 µm, B - 500 nm, C - 500 nm.

**Figure 6:**
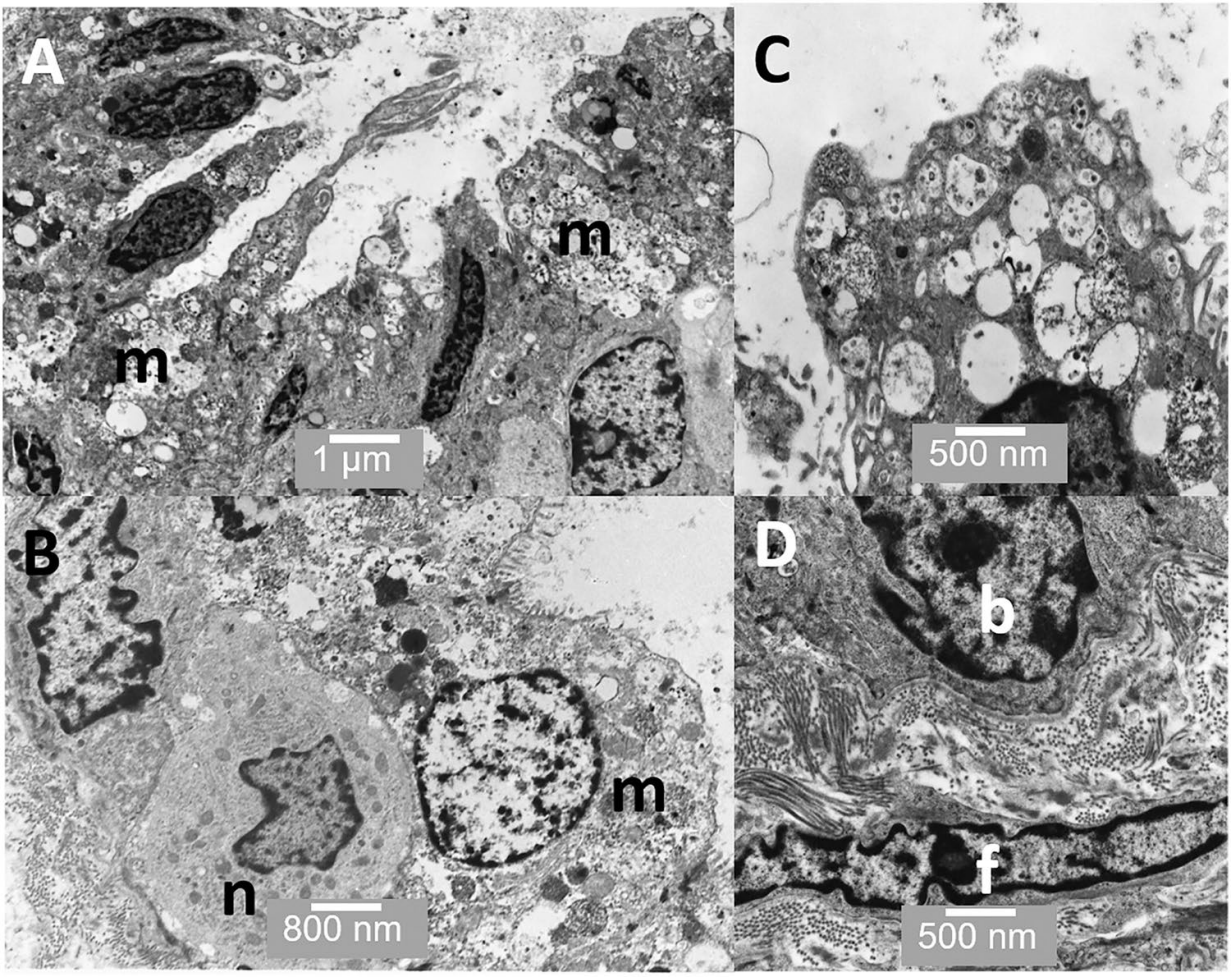
TEM through an acinus. (**A**) Shows several highly active merocrine secretory cells (m); (**B**) shows a neuroendocrine cell (n) below a merocrine secretory cell (m); (**C**) shows the apical region of one of the merocrine secretory cells filled with vesicles; and (**D**) shows a basal cell (b) and a fibroblast (f) in the stroma immediately below it. Scale bars: A - 1 µm, B - 800 nm, C - 500 nm, D - 500 nm.

**Figure 7:**
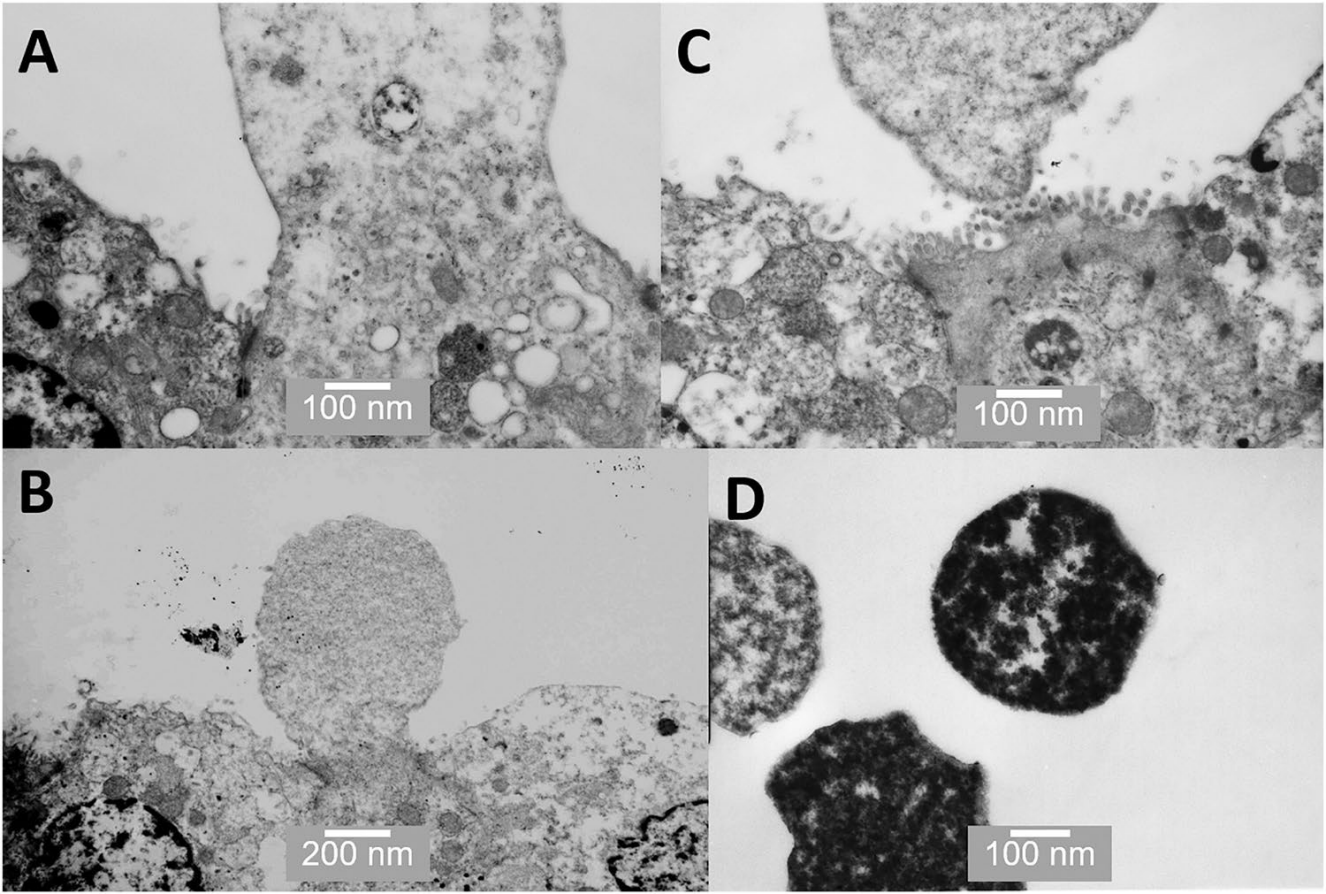
TEM images of various stages of apocrine secretion. (**A**) shows the protrusion of a dome from the apical surface; (**B**) shows the narrowing at the base of the dome to form a neck; (**C**) shows the separation of the aposome from the cell surface; and (**D**) shows the separated aposomes clearly containing some dense cytoplasmic material. Scale bars: A - 100 nm, B - 200 nm, C - 100 nm, D - 100 nm.

**Figure 8:**
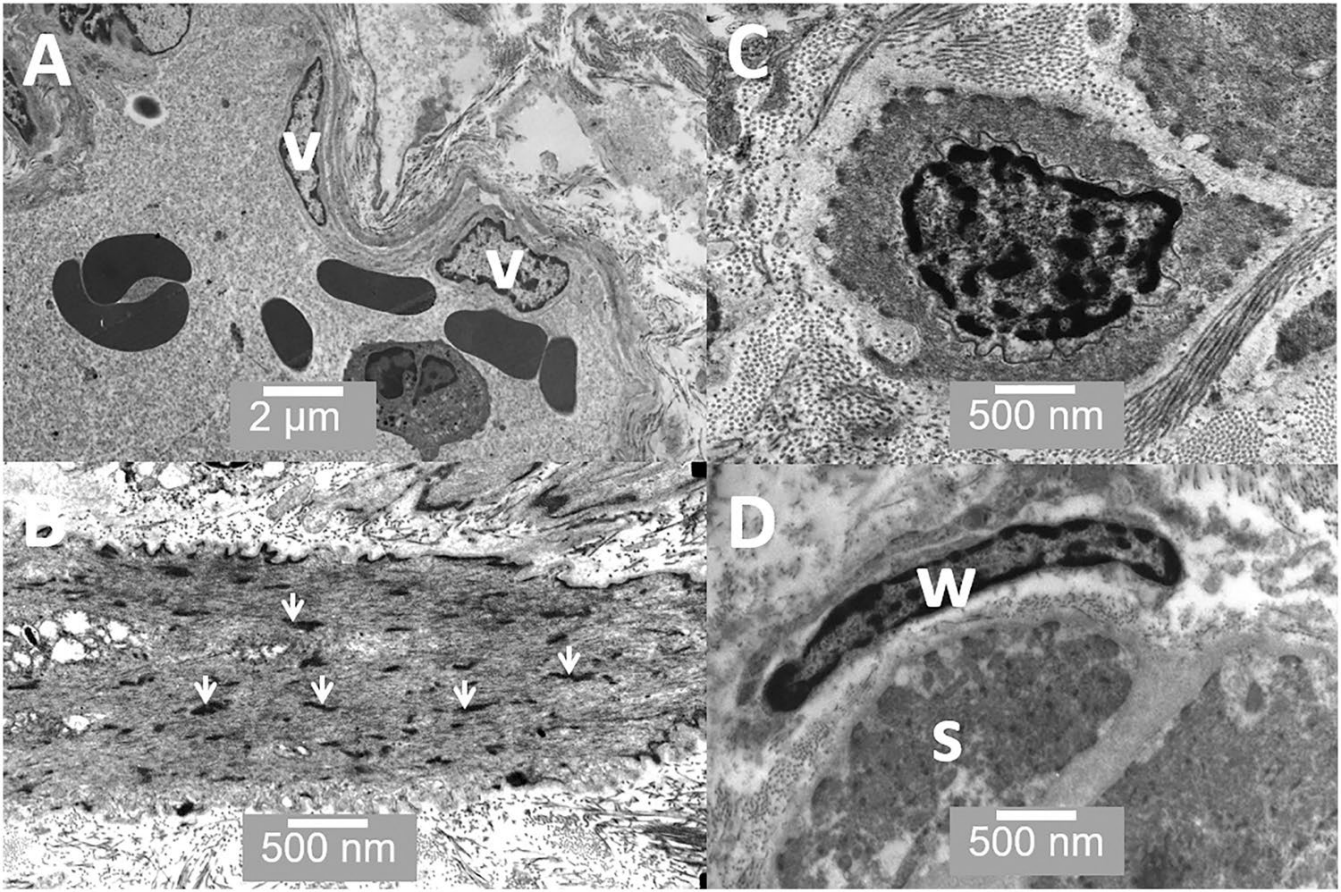
TEM images of the prostate stroma. (**A**) shows a minor blood vessel lined with vascular endothelial cells (v); (**B**) shows smooth muscle in longitudinal section, note the dense bodies (arrowheads) found in smooth muscle; (**C**) shows smooth muscle in cross-section surrounded by collagen bundles; and (**D**) shows a wrapping cell (w) surrounding a muscle bundle (s). Scale bars: A - 2 µm, B - 500 nm, C - 500 nm, D - 500 nm.

Figures 5–8 show TEM images of the prostate. Figure 5A shows some of the cell types present in acini including merocrine and basal cells. Figure 5B shows a putative basal stem cell and Figure 5C shows a novel cuboid epithelial-like cell from the ridge region. Interestingly, both the putative basal stem cell and the cuboid epithelial cell have low nucleus-to-cytoplasmic ratios. Figure 6A shows merocrine cells at low magnification; Figure 6B a neurosecretory cell next to a merocrine secretory cell and Figure 6C the apical region of a merocrine cell filled with secretory vacuoles (Figure 6C). Figure 6D shows a sub-epithelial fibroblast.

With images from the TEM, apocrine cells are much harder to definitively identify; as the 2-D nature of the sectioning process means that it is difficult to obtain sections showing the apocrine vesicle in the process of budding off. However, Figure 7 shows a reasonably convincing series of images including the narrowing of the neck region (Figures 7A,B), budding off (Figure 7C) and finally the released apocrine vesicles (Figure 7D).

Figure 8 shows a series of images from the prostatic stroma. Figure 8A shows a vascularised region very close to the secretory cells. Figure 8B also shows stromal smooth muscle in longitudinal section and Figure 8C stromal muscle in cross section. Figure 8D shows a wrapping cell associated with a muscle fibre; these cells have been identified in mouse but we were unable to find any published electron microscope images of these cells in human prostate.

The results from the Inductively Coupled Plasma Mass Spectrometry study of the prostate samples obtained from Caucasian men resident in NW England, revealed zinc levels of 54.7, 59.8, 109.0, 46.1, 40.2, 23.5, 45.7, 71.9, 92.6 and 143 mg/kg. X-ray microanalysis of 6 samples revealed the merocrine cells had mean percentage dry weight zinc content of 1.52% ± 0.47 and the prostasomes 0.05% ± 0.08. An unpaired t-test showed the difference to be highly significant *p* < 0.0001. Full details of the X-ray microanalysis protocol and the individual X-ray spectra are included in the Supplementary Information (SI) Figs S1–S3.

## Discussion

There are a number of cell types present within the acinar or alveolar structures in the human prostate. Firstly, the basal putative stem cells; these are present in the basal layer in the pseudostratified region. They are flat and elongated in direct contact with the basal lamina Their nuclei are often oval-shaped and they appear unspecialised with sparse cell organelles and they have a low nuclear-to-cytoplasmic ratio. Secondly, are the merocrine secretory cells, which are present in the pseudostratified region associated with the basal cells. These have a rounded apical surface with microvilli, a high nuclear-to-cytoplasmic ratio and have all the classic features of a merocrine secretory cell, including abundant rough endoplasmic reticulum (RER) and numerous vesicular structures within the cell. Thirdly, are the apocrine secretory cells; these are also present in the pseudostratified region in close association with basal cells and they are sometimes observed next to merocrine secretory cells. They are characterised by apical surface protrusions and contain far fewer cytoplasmic vesicles than are present in the merocrine secretory cells. Characteristically, they have a higher nuclear-to-cytoplasmic ratio than the merocrine secretory cells. Also present are low numbers of neuroendocrine cells.

**Figure 9:**
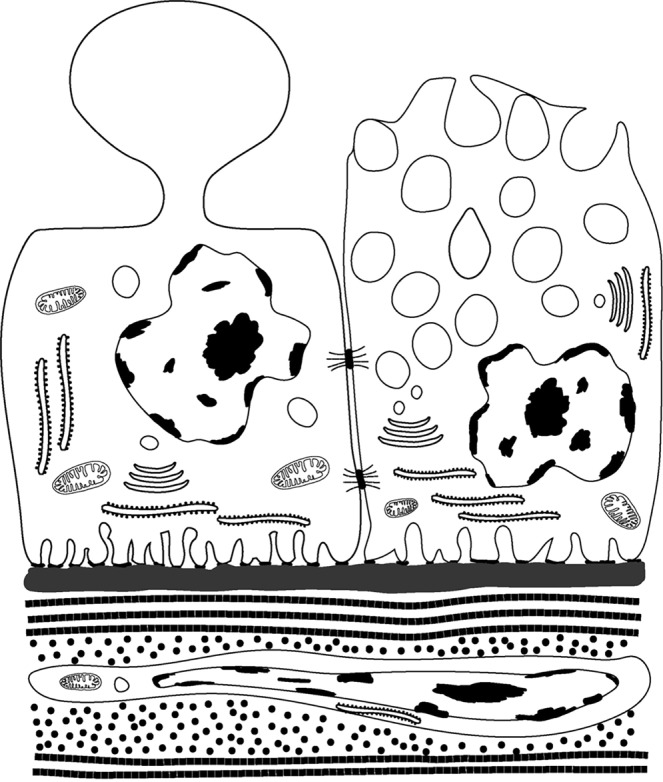
The illustration shows the differences between apocrine (left) and merocrine (right) secretory cells. Apocrine cells are characterised by dome-like protrusions, which pinch off and are released into the lumen. The vesicles in merocrine secretory vesicles are derived from the RER and Golgi apparatus and the contents released *via* the fusing with the apical membrane and exocytosis. The bottom of the illustration shows a sub-epithelial stromal fibroblast surrounded by collagen fibrils.

**Figure 10:**
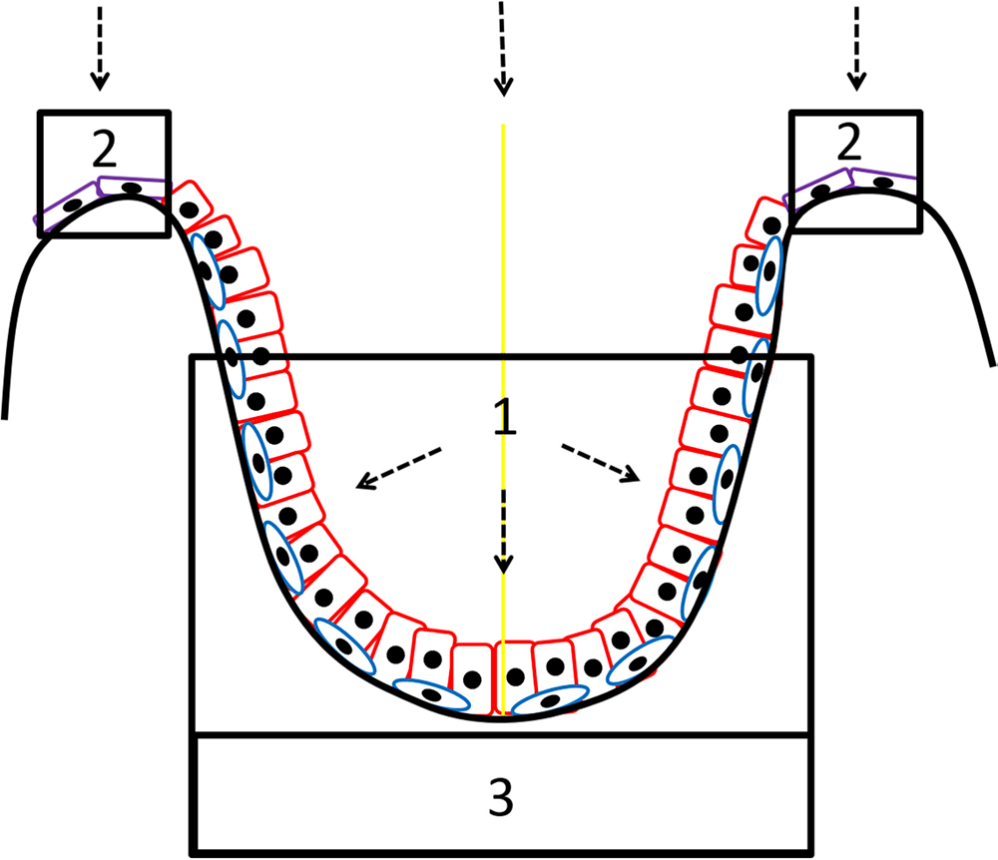
This micrograph orientation figure shows a greatly simplified diagram of a 2-D cross section through the centre of an acini with 3-D symmetry about the yellow line. The perspective of the TEM 2-D micrographs is the same as in this 2-D diagram. The perspective of the SEM micrographs should be visualised as being from the origin of the dotted arrows. Figures 1, 2, 3, 5A,B, 6 and 7 are from the area labelled 1. Figures 4 and 5C are from the areas labelled 2. Figure 8 is from the area labelled 3. Secretory cells are shown as red, putative basal stem cells as blue and the non-secretory cuboid epithelial cells as purple.

Merocrine secretory cells within the prostate have been extensively studied; in conventional merocrine secretion, the proteins are produced in the RER before passing though the Golgi and into secretory vesicles, which fuse with the apical cell membrane and release their contents into the lumen as shown in the cell on the right in Figures 9 and 10. Merocrine secretion is generally credited with producing the vast majority of the best-characterised prostatic secretions, including proteolytic enzymes, prostatic acid phosphatases and prostate specific antigen (PSA). In merocrine secretion, the secreted protein has a signal peptide that facilitates transfer of the peptide through the RER membrane.

Our ICPMS data confirms the high levels of zinc in hydrated samples but at the same time, we were able to detect significant levels of zinc in merocrine cells but only trace amounts in some prostasomes. We employed X-ray microanalysis to analyse individual merocrine cells and prostasomes *in situ*. Zinc has previously been detected in prostate tissue using X-ray microanalysis^11^, but individual prostasomes have not previously been analysed using this method. X-ray microanalysis has been reported to have a detection limit of between 10 ppm and 1000 ppm^12^ depending on the element and the topology of the samples. The topology of our samples is not optimal but our results show that if zinc is present in the prostasomes it is present at a lower concentration than 1 mg/g (0.1%) dry weight. Since levels of 3.1 mg/g dry weight have been reported in seminal fluid^13^ and since our merocrine cells had on average >10 times higher zinc levels than the prostasomes, our findings support the consensus that the primary mechanism for zinc secretion into the prostatic fluid is *via* merocrine cells.

It is known that the secretion of zinc involves a number of transporter Zip proteins. Zip1 is present in the basal membrane and Zip2 and Zip3 in the apical membrane; these Zips import zinc into the cell. ZnT2 is found in the endoplasmic reticulum, while ZnT4, ZnT6, and ZnT7 are present in the Golgi apparatus; these ZipTs make up the zinc secretory pathway^14^. The localisation of the ZnTs in the ER and Golgi support the hypothesis that zinc is released *via* merocrine secretion. Hydrated prostatic fluid is reported to contain ∼500 μg Zn/mL fluid in stark contrast with the 1–2 μg Zn/mL present in serum^14^. The physiological role(s) of such massive levels of zinc are still not fully understood^14^. However, since the prostate secrets large amounts of citrate, it has been proposed that a high level of zinc in prostate cells facilitates this by blocking the enzyme m-aconitase and, thus, inhibiting citrate oxidation^15^. A number of studies have correlated zinc concentration in semen plasma with sperm quality^16^. Other explanations proposed include possible roles as an antioxidant, an antibacterial agent^17,18^ or in helping maintain the stability of sperm^19^.

Although the existence of apocrine secretory cells in the prostate is generally accepted, little is known about their function in prostate. In other words, why is apocrine secretion needed in the prostate? Apocrine secretion is relatively rare in the body; apocrine cells have a unique secretory mechanism where at the point of release the apical region of the cell becomes “pinched off” or “decapitated” (Figures 2C and 7A–D). This results in the release of a large membrane-bound vesicle or aposome into the lumen. Apocrine secretory cells are uncommon but can be found in the skin (especially round the armpits and pubic areas), the external auditory canals, on the eyelids and the breast (where they produce colostrum during parturition). Apocrine secretion, unlike the merocrine pathway, does not involve the conventional RER, Golgi apparatus, and secretory vesicle pathway. The proteins and other molecules released during apocrine secretion do not require a peptide signal sequence for passage through the RER membrane but instead, are produced in the cytoplasm and then transported to the apical region of the cell, which then buds off. The process of apocrine secretion is illustrated in Figure 9 by the cell on the left.

While it is accepted that apocrine secretion occurs within the prostate, its specific role and importance herein remains unclear. Our observations confirm that apocrine secretory cells actually make up a significant proportion of secretory cells in the prostate and that they appear very active. For the first time, we have SEM images of the actual pinching of the neck region and the release of the aposome (Figure 2C). In our view, prostasomes are key to our understanding of the role of apocrine secretion. The term prostasomes is generally used to classify the extracellular vesicles released into prostatic fluid by prostate epithelial cells. Prostasomes are larger than exosomes, which are cell-derived vesicles that are found in many biological fluids, including blood and urine and are usually <50 nm in size. The prostasomes we have observed range in size from 150 nm to over a micron. They have been proposed to have a remarkable number of properties including facilitating sperm mobility, semen calcium balance, semen liquefaction, complement inhibition, immunosuppression, suppression of infection and HIV transmission and several others^20,21^. Some workers have hypothesised that prostasomes arise through exocytosis or diacytosis and not through apocrine secretion. For example, Ronquist and Nilson (2004) state that they feel it is unlikely that prostasomes could be released *via* apocrine secretion^22^. We would challenge this posit that a large proportion of prostasomes are generated *via* apocrine secretion. Our investigation clearly shows that the apocrine cells are releasing large membrane bound vesicles or prostasomes (Figures 2C and 7A–D) in very large numbers (Figure 3C). These are far larger, ranging from 150 nm to over a micron in diameter (Fig. 2D), than the small vesicles (<50 nm) sometimes observed on the surface of merocrine cells (Figure 3A), which may be secretory vesicles or exosomes. It is also worth mentioning that the size of these prostasomes as shown in our images is likely to be an underestimate because of the shrinkage, which takes place during processing for SEM^23^.

Recent studies have shown that a very large number of proteins are present in prostasomes, with one study reporting nearly 139 proteins^24^ and a second 440^25^. Critically many of the proteins identified in these studies do not have conventional signal peptide sequences; therefore, there is no conventional pathway for them to be released into the lumen of the prostate. Examples of these proteins include the Annexins; other proteins that stand out are: dynein, actin A, actin Bα and β-tubulin. One study reports the presence of glycolytic enzymes^26^. There are even reports of prostasomes containing chromosomal DNA^27^.

To us, it seems clear that the sheer volume of proteins and other molecules found without signal peptide sequences within prostasomes can best be explained if they are generated *via* apocrine, not merocrine secretion. This also explains why apocrine cells are needed within the prostate. With regard to why apocrine secretion releases such a diverse range of molecules, which are not normally secreted; again, there is a logical explanation. It has been pointed out^28^ that as spermatids leave the testis to enter the epididymis they have very limited biosynthetic capacity and for maturation they depend on the transfer of secreted products from epididymal epithelial cells and that these are transferred by epididymosomes, membrane vesicles produced epididymal cells *via* an apocrine secretion mechanism. It seems to us highly probable that the aposomes produced by the apocrine prostate cells have a very similar role. Since in mature mammalian sperm transcription is closed down and it can be days to weeks before the sperm are released it would make sense to have some mechanism for the replenishment of key proteins at the point of their release. For example, the enzymes needed for glycolysis or proteins associated with the sperm flagella have both been reported in human prostasomes^26^. Transfer of these proteins into the cytoplasm to the sperm at point of release *via* an apocrine-secreted prostasome would be of clear benefit to mobility and such secretion allows large complex proteins without signal peptide sequences to be transferred in a fashion that merocrine secretion does not facilitate. Thus, we propose that it is apocrine secretion, not conventional merocrine secretion, which is primarily responsible for the generation of prostasomes-containing proteins without conventional signal peptide sequences.

In addition to the previously described cell types, this investigation also identified a novel cell type, namely a polygonal cuboid epithelial cells (Figures 4C,D) present as a monolayer in the ridge regions (Figures 4A,B) between the acini. These are polygonal in shape showing very well defined cell junctions with a flat apical surface and with very small or no microvilli (Figures 4C,D). They have a low cytoplasm-to-nuclear ratio (Figure 5C). These cells are clearly distinct from the secretory or basal cells. They are different from the basal epithelial cells in that they are not directly associated with secretory cells and form a monolayer, rather than a pseudostratified layer like the basal and secretory cells. In addition, unlike the basal cells in the acinar regions they are in direct contact with each other and form a flat featureless surface with very distinct cell borders. They also appear to be non-secretory in function (Figures 4C,D and 5C). Interestingly, these epithelial cells reside in a different environment to that of the basal cells in the acinar regions; they are in the ridges separating the acini (Figures 4A,B).The location of these cells relative to the other cell types reported in this paper can be seen in Figure 10. Clearly, these epithelial cells must be maintained and replenished by a stem cell population. We see possibilities; the first is that epithelial cells are produced by the basal cells in the acinar regions and that transit-amplifying or progenitor cells migrate upwards out of the acini onto the ridge structures, which separate the individual acini. There is also a second more speculative possibility; namely, that epithelial cells on the ridges harbour a separate stem cell population and that these produce basal or progenitor cells which migrate downwards into the acini and produce the terminally-differentiated secretory cells. Most studies looking at prostate have been done using the 2-D techniques of light microscopy or TEM; our experience has shown it is almost impossible for us to identify this population of cells using these 2-D methods. It is only when using SEM that it becomes clear that they are a morphologically distinct and spatially defined cell type. It is worth mentioning that stem cell populations in tissues tend to be in regions, which are spatially away from the more differentiated cell types; there are many examples including the limbus of the cornea and the base of the hair shaft in skin^29,30^.

Our observations of the prostate stroma are mostly in line with previous reports. The stroma is composed mostly of bundles of collagen fibrils of a reasonably consistent diameter (≈30 nm). Between collagen bundles are a sparse population of fibroblast cells. These fibroblast cells appeared as flattened elongated cells often running parallel to the basal lamina, some are within a few 10’s of nanometres of the basal lamina of the glandular cells (Figure 6D). There are a variety of sizes of blood vessels (Figure 8A), some running very close to the basal lamina of the glandular cells The mechanisms for the maintenance and renewal of stromal cells in the prostate remains unclear but recent observations in mouse, using immunohistochemistry, have reported four distinct stromal subtypes^10^. These include not just conventional fibroblast cells, but also sub-epithelial fibroblast cells located between the basal lamina and smooth muscle cells (Figures 8B,C) and so called wrapping cells; these are fibroblast-like cells intimately associated or “wrapping around” smooth muscle bundles. Herein, we provide the first TEM image of a wrapping cell from human prostate (Figure 8D). There is likely to be sonic hedgehog (SHH) signalling from prostate basal epithelial cells to adjacent stromal cells and the SHH-target gene *Gli1* is preferentially expressed in sub-epithelial fibroblast cells^31^. Interestingly, it has been suggested that both the sub-epithelial fibroblasts and wrapping cells in mouse may be replenished by distinct or different stem/progenitor cells. In mouse, the smooth muscle cells are probably replenished from the existing smooth muscle cell population^10^. Our observations are consistent with this theory, as we observe that in human prostate, the sub-epithelial fibroblasts are spatially within a few hundred nanometres of the basal epithelial cells and so would receive high levels of  SHH produced by the basal cells.

### Summary

This paper shows images of all the stages of apocrine secretion and the production of apocrine prostasomes. We present evidence that these apocrine-derived prostasomes make up a significant component of the prostasomes secreted and posit why apocrine production of prostasomes is critical for sperm viability. We also identify a previously unreported epithelial cell type present as a monolayer along the ridges between the acinar structures and present images of the cell types present in the stroma including the sub-epithelial fibroblast, smooth muscle cells and wrapping cells.

## Supplementary information

Supplementary Info
