# Supplementary material for: An analysis of benign human prostate offers insights into the mechanism of apocrine secretion and the origin of prostasomes
Source: Sci Rep. 2019 Mar 14;9:4582. doi: 10.1038/s41598-019-40820-2 (PMC6418221; doi:10.1038/s41598-019-40820-2)
Supplement: Supplementary file 1 — Supplementary Info [file 41598_2019_40820_MOESM1_ESM.pdf]

Supplementary Information File for:

An analysis of benign human prostate offers insights into the mechanism of apocrine secretion and the origin of prostasomes

Nigel J Fullwood*[1], Alan J Lawlor[2], Pierre L Martin-Hirsch[3], Shyam S Matanhelia[3], Francis L Martin[4]

[1]Biomedical and Life Sciences, Faculty of Health and Medicine, Furness College, Lancaster University, Lancaster LA1 4YG, UK; [2]Centre for Ecology and Hydrology, Lancaster Environment Centre, Library Avenue, Bailrigg, Lancaster LA1 4AP, UK; [3]Sharoe Green Lane North, Royal Preston Hospital, Fulwood, Preston PR2 9HT, UK; [4]School of Pharmacy and Biomedical Sciences, University of Central Lancashire, Preston PR1 2HE, UK.

Supplementary Information X-ray Microanalysis

Analysis was carried out with a JEOL JSM-7800F high performance Field Emission SEM fitted with a high performance X-ray Energy Dispersive Spectrometer (X-Max50) with a large area 50 mm^2 Silicon Drift Detector (SDD) from Oxford Instruments. The prostasomes and merocrine cells were analyzed for zinc *in situ* within the prostate by focusing the electron beam on individual prostasomes or merocrine cells and collecting the characteristic X-rays produced. Analysis of the X-ray counts was carried out with Aztec version 3.0 (Oxford Instruments).

X-ray microanalysis was from 6 samples for each sample spectra were collected from individual merocrine cells and prostasomes. The analysis was done at 10 KV, with a 2-min collection time, total X-ray counts for each analysis varied between 190,000 and 260,000.

The spectra shown in this Supplementary Information and used for the t-test were selected on the basis of the following criteria: 100% confidence in identification of the structure analysed as a prostasome or merocrine cell, quality of ultrastructural preservation, no specimen drift observed during the 2 minute collection time, no evidence of damage from the electron beam after analysis, no evidence of the X-ray detector being blocked from area of analysis by sample topology, at least 150,000 X-ray counts obtained from the area analysed.

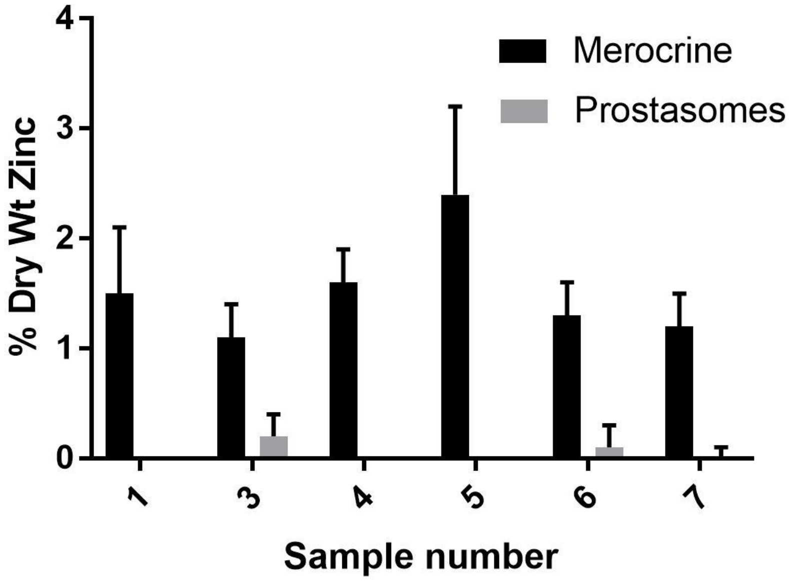

Figure S1. Histogram showing levels of zinc in merocrine cells and prostasomes as % dry weight from 6 samples. There is no sample 2 as the spectra from sample 2 did not meet the inclusion criteria. The standard deviation bars are calculated from the X-ray counts collected in the 2-minute collection time. Total counts ranged between 190,000 and 260,000 counts.

Supplementary Information X-ray microanalysis spectra

Several elements in the spectra are derived from specimen preparation protocols these include gold and copper which originate from the sputter target used to coat the sample, osmium tetroxide fixative used to maintain the integrity of the membranes during processing. The presence of trace amounts of Iodine in some samples may be genuine or due to contamination from the povidone–iodine solution used during surgery. We know of no possible sources of contamination by zinc either during surgery or during preparation of the samples for X-ray microanalysis.

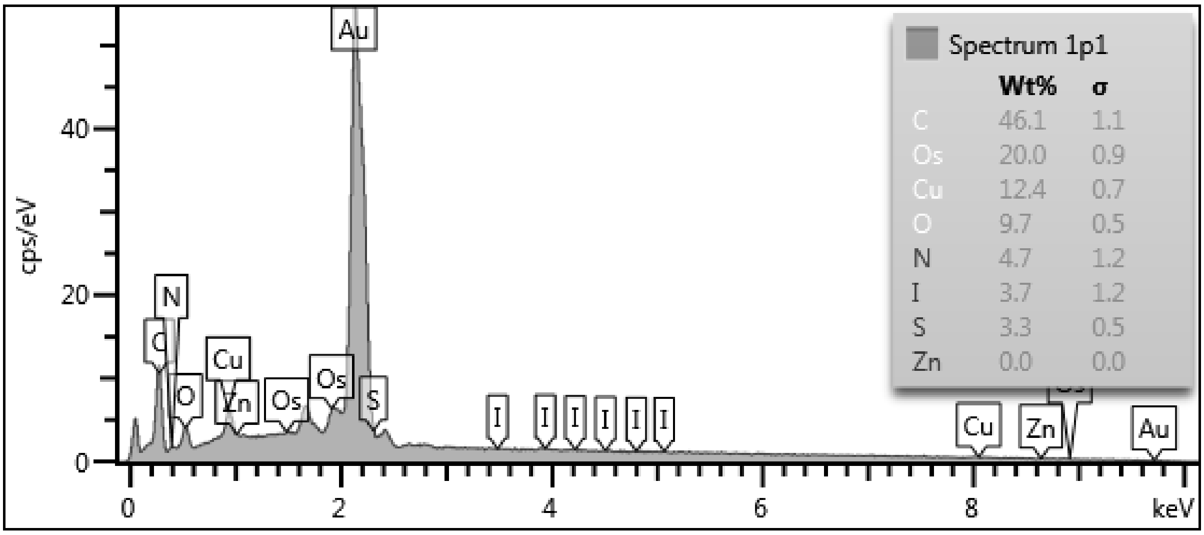

Figure S2a. X-ray microanalysis spectra of Sample 1 prostasome

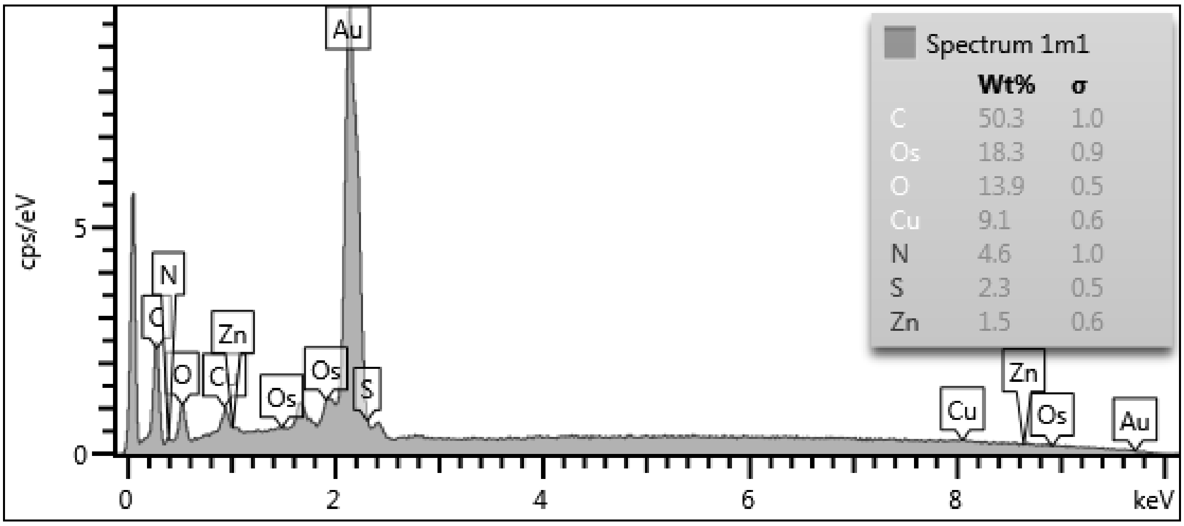

Figure S2b. X-ray microanalysis spectra of Sample 1 merocrine cell

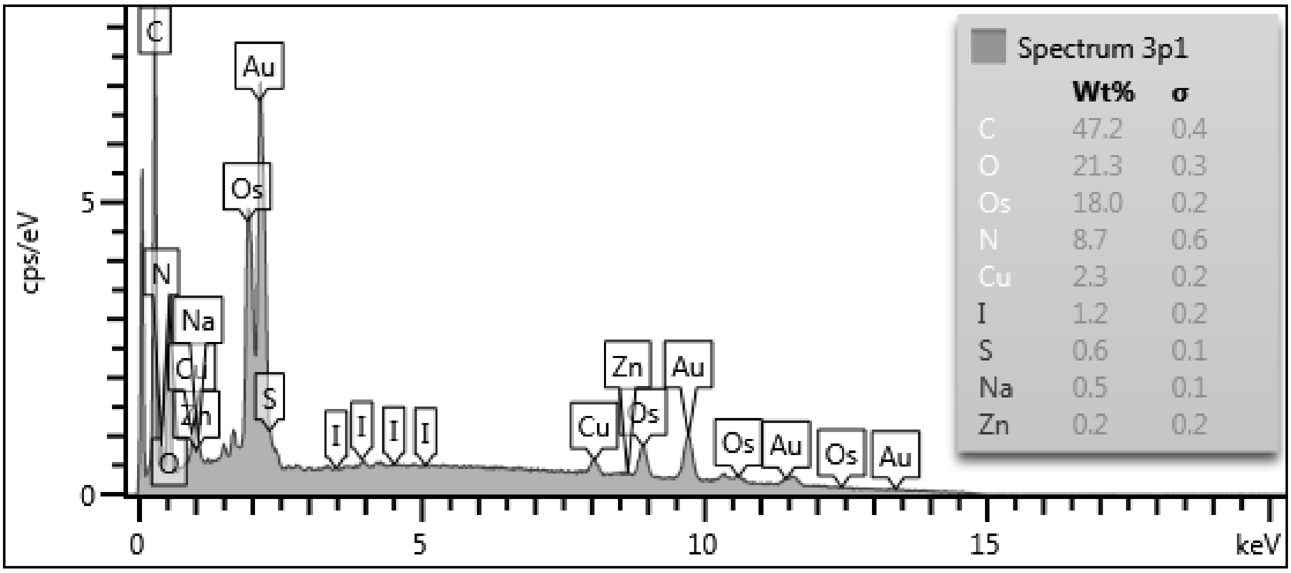

Figure S2c. X-ray microanalysis spectra of Sample 3 prostasome

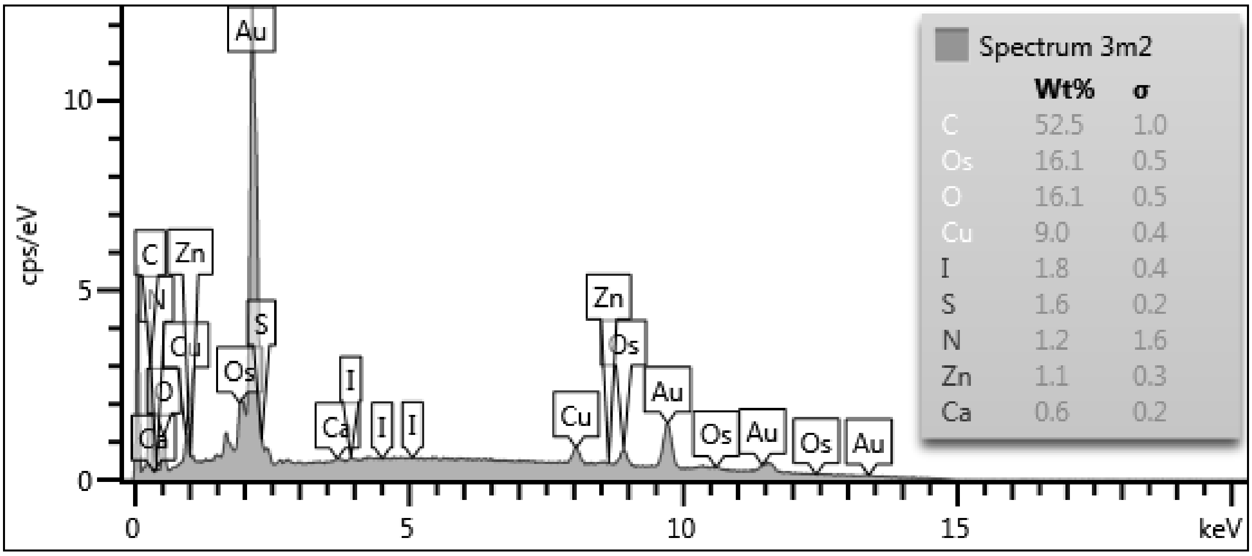

Figure S2d. X-ray microanalysis spectra of Sample 3 merocrine cell

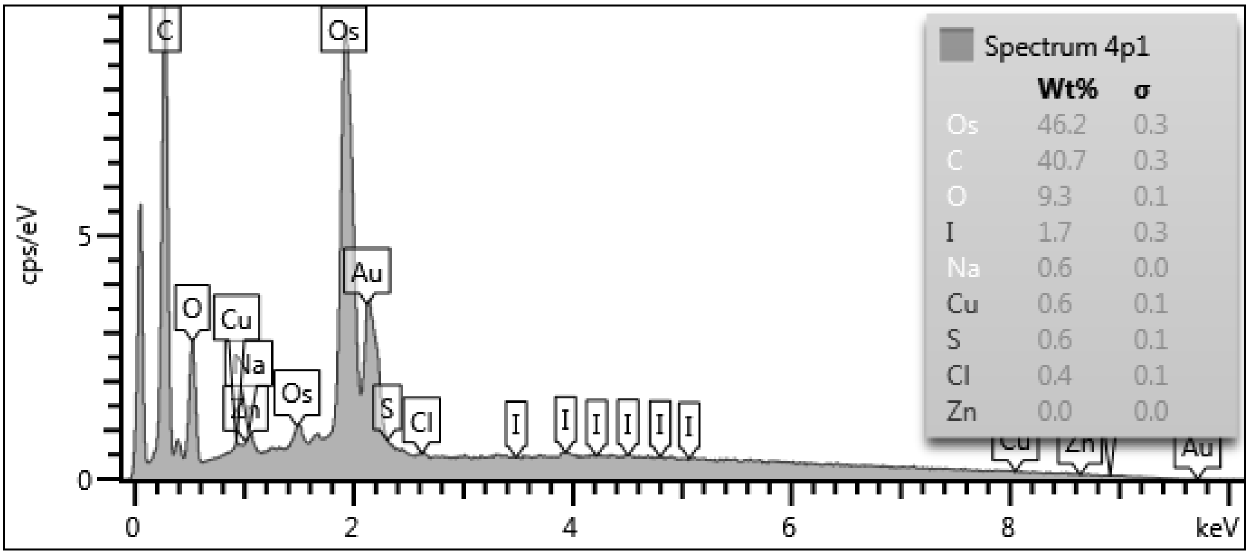

Figure S2e. X-ray microanalysis spectra of Sample 4 prostasome

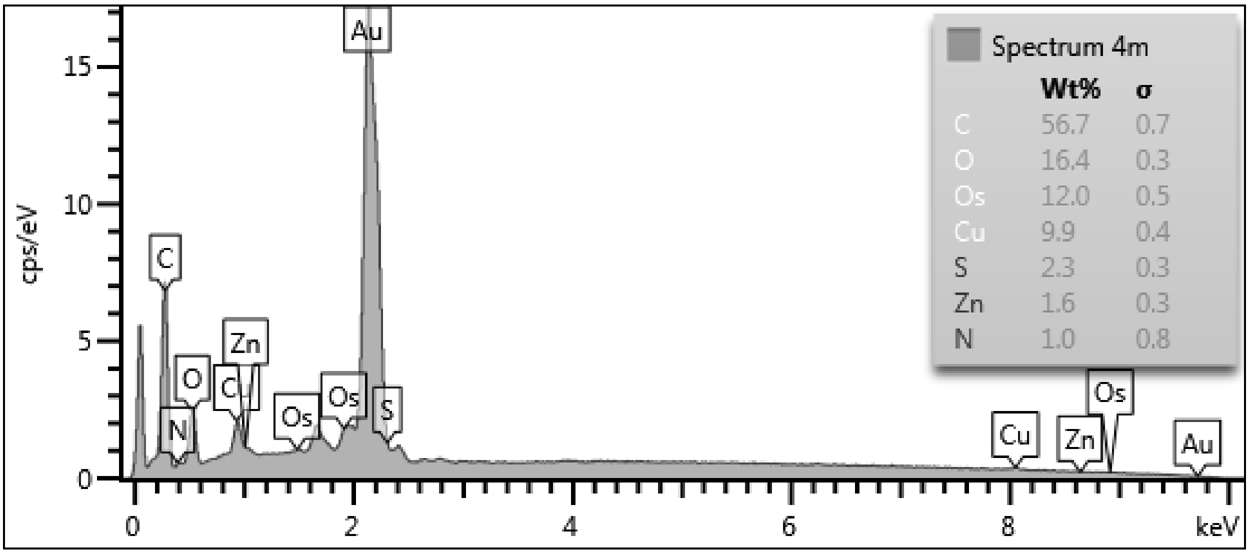

Figure S2f. X-ray microanalysis spectra of Sample 4 merocrine cell

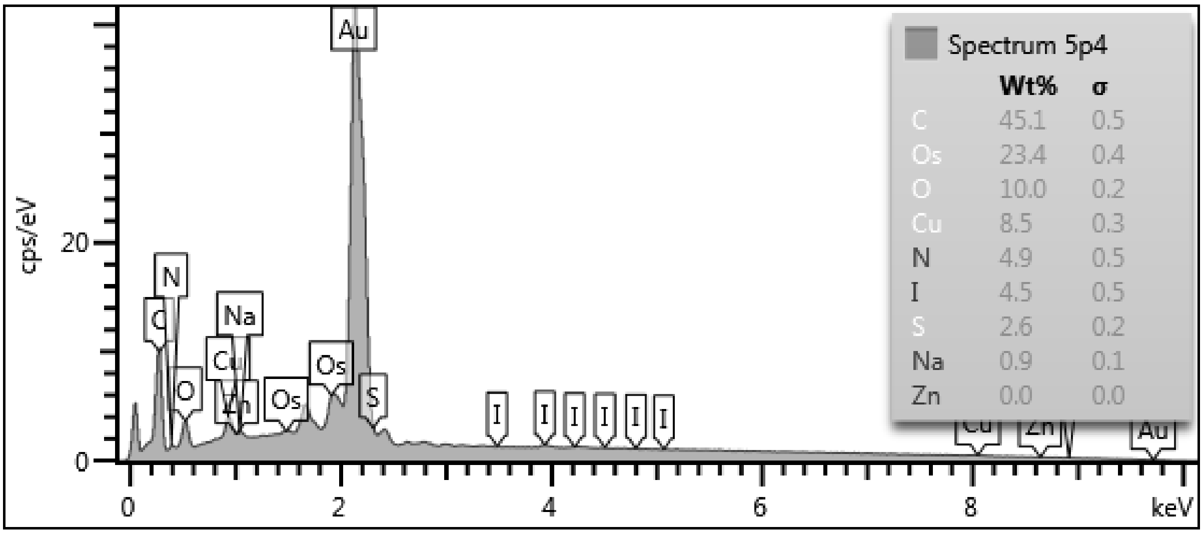

Figure S2g. X-ray microanalysis spectra of Sample 5 prostasome

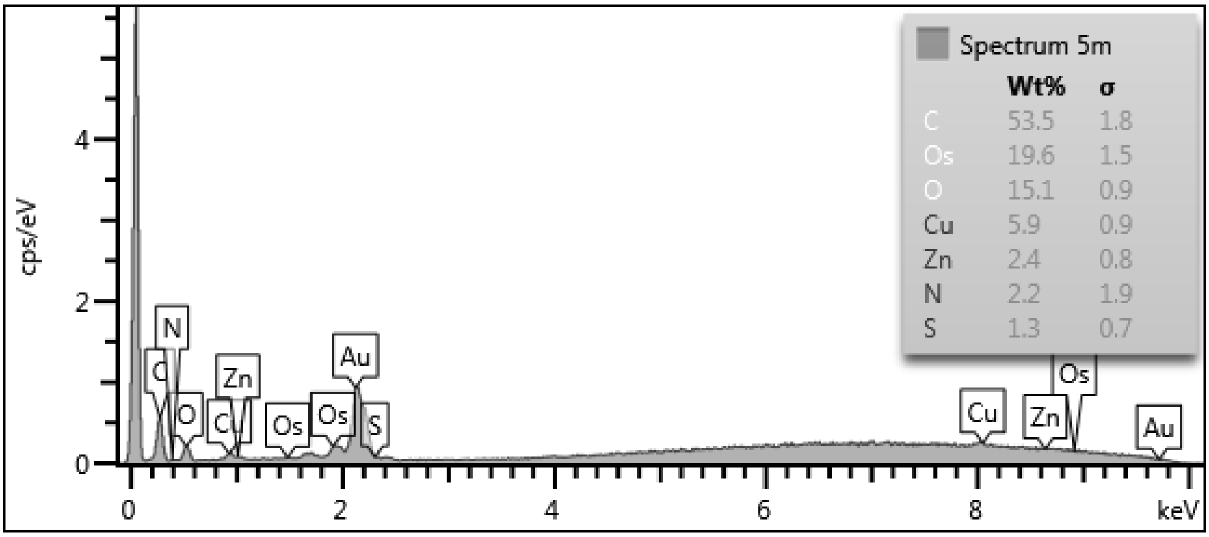

Figure S2g. X-ray microanalysis spectra of Sample 5 merocrine cell

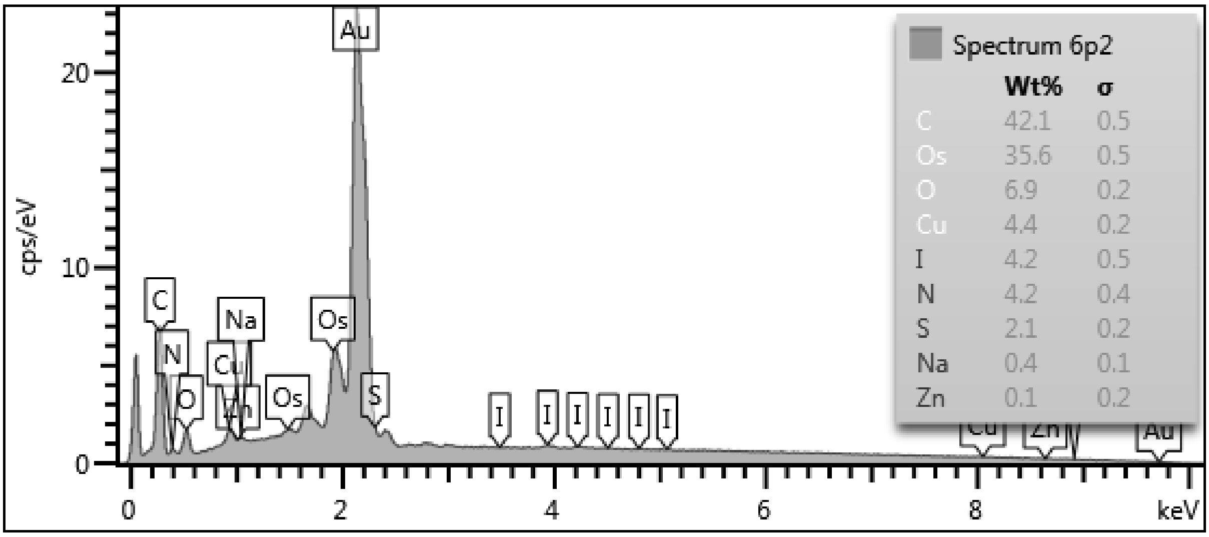

Figure S2h. X-ray microanalysis spectra of Sample 6 prostasome

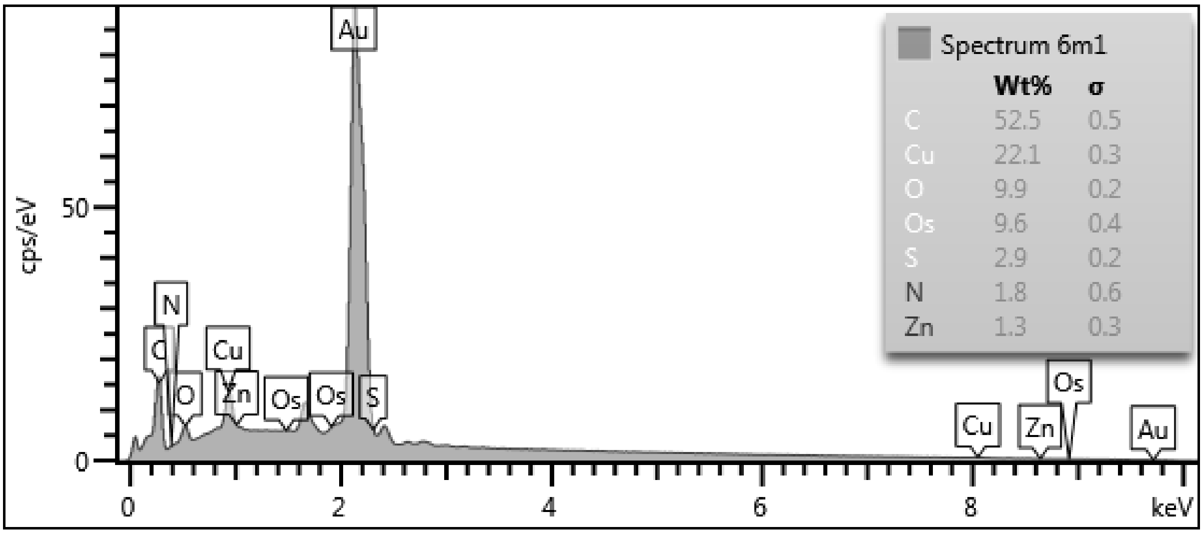

Figure S2i. X-ray microanalysis spectra of Sample 6 merocrine cell

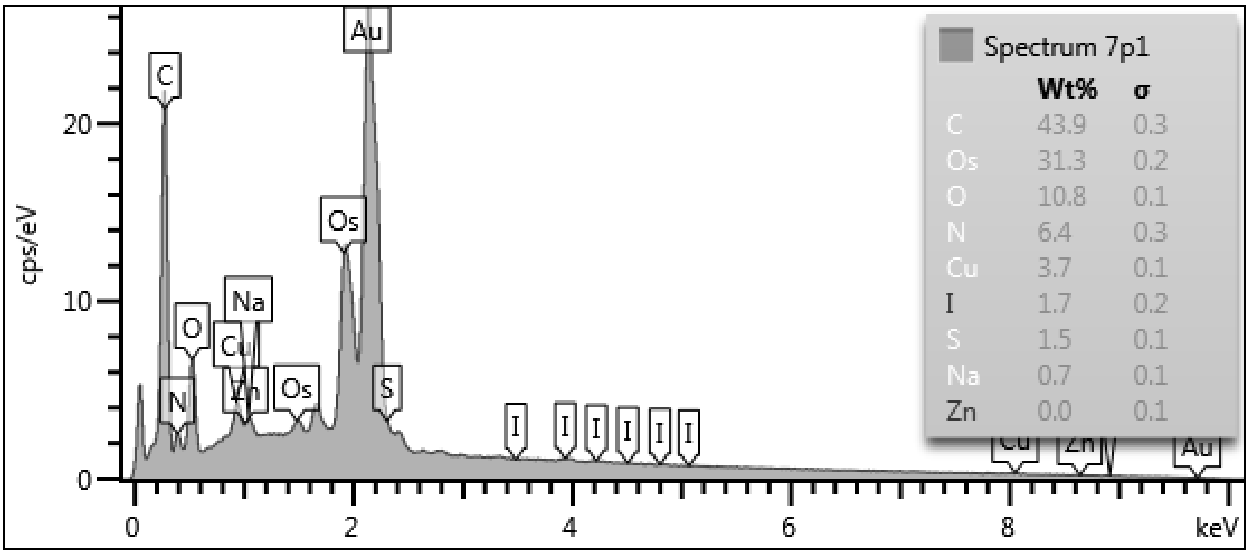

Figure S2j. X-ray microanalysis spectra of Sample 7 prostasome

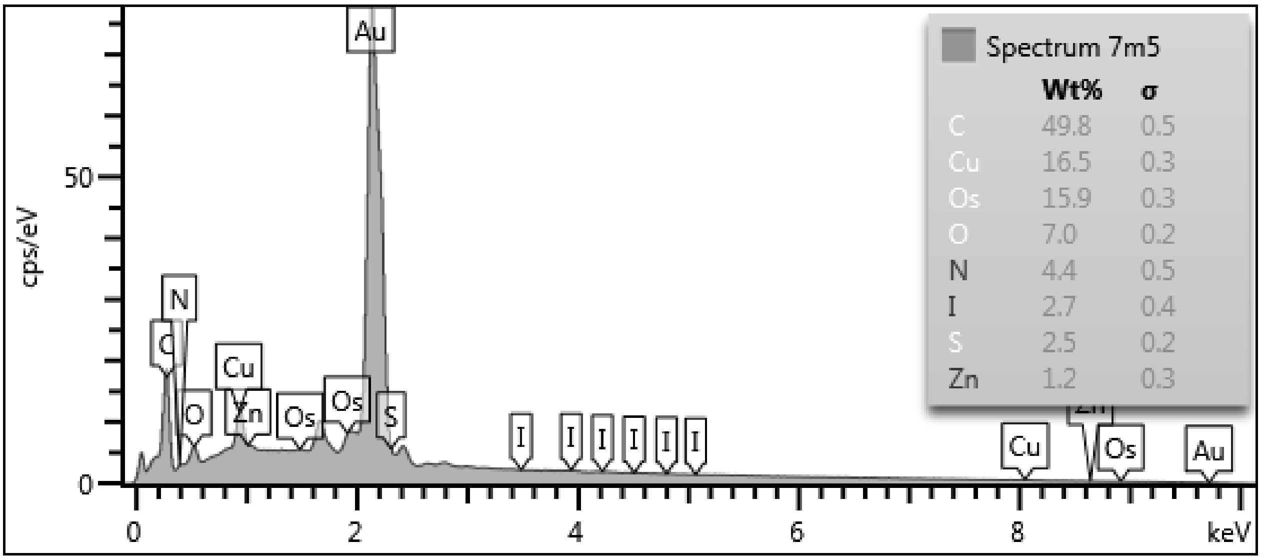

Figure S2k. X-ray microanalysis spectra of Sample 7 merocrine cell

Supplementary Information SEM images

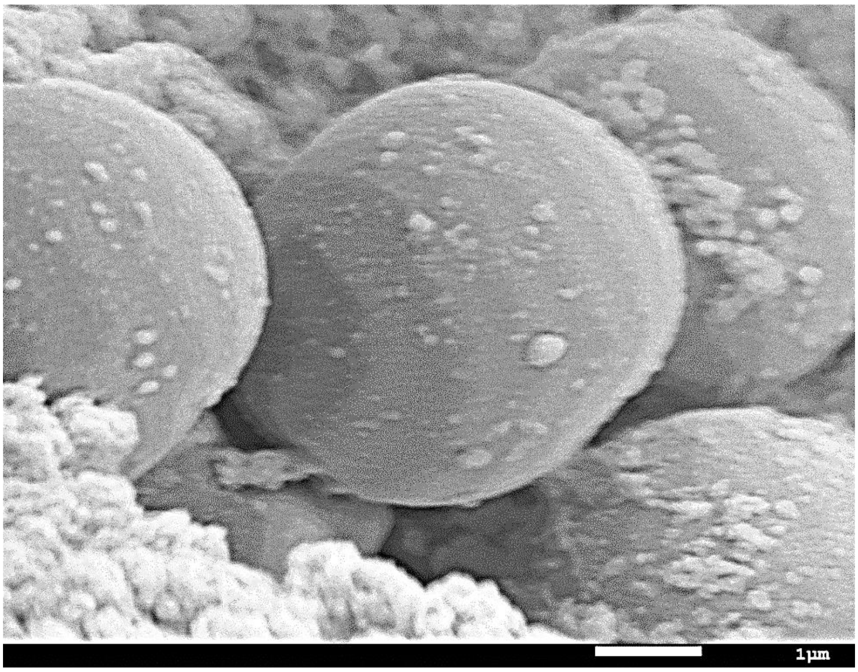

Figure S3a. SEM showing it is possible to locate prostasomes over a micron in diameter making it feasible to carry out elemental X-ray microanalysis on individual prostasomes

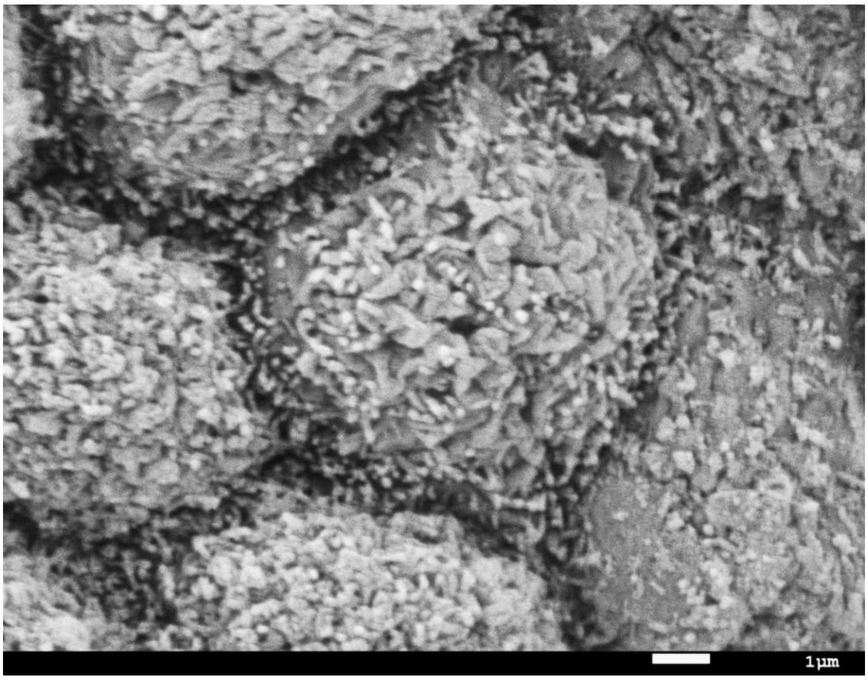

Figure S3b. SEM showing it is possible to clearly identify merocrine cells making it feasible to carry out elemental X-ray microanalysis on the apical region of individual merocrine cells.
